# Decision-making capacity evaluations: the role of neuropsychological assessment from a multidisciplinary perspective

**DOI:** 10.1186/s12877-020-01932-x

**Published:** 2020-12-10

**Authors:** Sarah Wood, Klaus Bally, Christine Cabane, Patrick Fassbind, Ralf J. Jox, Thomas Leyhe, Andreas Monsch, Manuel Trachsel

**Affiliations:** 1 U.S. Army Behavioral Health Clinic, Wiesbaden, Germany; 2 Center for Primary Health Care, University of Basel, Basel, Switzerland; 3 Kindes-und Erwachsenenschutzbehörde Baselland, Liestal, Switzerland; 4 Kindes-und Erwachsenenschutzbehörde Basel-Stadt, Basel, Switzerland; 5 Palliative and Supportive Care Service and Institute of Humanities in Medicine, Lausanne University Hospital, and University of Lausanne, Lausanne, Switzerland; 6 Department of Geriatric Medicine Felix Platter Hospital, University of Basel, Basel, Switzerland; 7 Institute of Biomedical Ethics and History of Medicine, University of Zürich, Zürich, Switzerland; 8 Clinical Ethics Unit, University Hospital of Basel, and University Psychiatric Clinics Basel, Basel, Switzerland

**Keywords:** Decision-making, Cognition, Capacity evaluation, Autonomy, Informed consent

## Abstract

Decision-making capacity (DMC) in aging adults has become increasingly salient as the number of older adults, life expectancy, and the amount of wealth to be transferred from older generations have all increased. The accurate and reliable determination of older adults’ DMC is a particularly important topic given its implication in legal, financial, and health decisions. Based upon the four-ability DMC model promulgated by Appelbaum and Grisso in the 1980’s, a number of MacArthur Competence Assessment Tools have been developed and widely utilized. However, these tools do not include cognitive testing or other sources of objective data and have limited validity in a medico-legal setting, necessitating additional options for the evaluation of DMC. This is significant from the perspective of the patient because they have a vested interest in accurate and objective assessment of their DMC across domains.

Given the disparities in the assessment of DMC, the authors propose, through this debate article, that the evaluation of DMC in the aging adult population utilize a combination of traditional interview and domain specific instruments and neuropsychological testing. To achieve a consensus on the issue, medical experts in a number of fields related to capacity evaluation, including psychiatry, neurology, neuropsychology, and general medicine were consulted and recruited as authors. Experts in Swiss law and ethics were also consulted and provided input.

A tendency to focus on a single capacity, and in particular, the ability to consent to medical treatment, arose in the literature. Similarly, there are many instruments purporting to evaluate a single capacity (e.g., consenting to medical treatment, managing finances), while other areas important to the evaluation of DMC received little attention (e.g., activities of daily living, the ability to live independently, to marry, to resist undue influence, and to make a will or advanced care directive). Medical and legal experts in the multidisciplinary group agreed that there is a clear need for more consistency across evaluation of DMC domains and that a combined approach of traditional methods and neuropsychological testing provides a more thorough evaluation and better serves the patient.

## Background

The issue of DMC evaluation has become increasingly relevant and will continue to gain importance as the world’s aging population and the number of people with dementia both increase. According to the World Health Organization, around 50 million people are affected by dementia and it was recently recognized as an international public health priority which spurred the creation of a Global Action Plan [1]. With an increased aging population comes an increase in disability, potential for financial and personal abuse, and need for support. The evaluation of older adults’ DMC is a particularly important topic given the significant legal, financial, and health ramifications. Additionally, the modern view of Advance Care Planning (ACP) is that of “a continuous, dynamic process of reflection and dialogue between an individual, those close to them, and their healthcare professionals” [2]. As such, it can be argued that DMC will not only need to be evaluated on a case-by-case basis, but repetitively in the course of a progressive dementia [2].

At present, there are limited standardized means for clinicians to evaluate an individual’s capacity to make important life decisions such as executing a will, consenting to medical treatment, or engaging in financial or highly personal contracts. Research to date has generally been domain-specific, including financial capacity [3], testamentary capacity, and consent for medical treatment. Several evaluation instruments have been developed to evaluate these specific domains of interest, such as the MacArthur Competence Assessment Tool for Treatment (MacCAT-T) [3], but are not entirely consistent with each other and do not generalize beyond a single capacity. Although it is agreed that development of a global “capacimeter” as described in the 1990’s [4] is too far-reaching, an increase in standardization of civil capacity evaluation could assist in the collaboration between professions [5] and provide better patient care overall.

In addition to psychiatrists and clinical psychologists, neuropsychologists are increasingly being consulted in medico-legal settings, including cases involving civil DMC [5]. The significant contribution neuropsychological assessment can make in this context has been highlighted in recent years, both in specific domains such as testamentary capacity, [6] and more broadly [7]. Neuropsychological performance, defined here as standardized scores obtained on measures of cognitive functioning, has been shown to strongly relate to current and future DMC and neuropsychologists are particularly equipped to assist in identifying which of the relevant areas may be impaired [8]. Accordingly, there is “a critical need for decision-making science to inform the design of capacity measures to reflect the multiple neuropsychological processes contributing to these decisions” [8 p274].

Additionally, recent research has identified cognitive impairment as the biggest challenge for physicians who have to evaluate DMC [9]. The majority of DMC evaluations concerns older adults and, importantly, psychiatrists and other medical health providers judge their training in capacity evaluation as insufficient [9]. According to the American Psychiatric Association’s Diagnostic and Statistical Manual of Mental Disorders, neurocognitive impairment is divided into mild and major, both of which require evidence of cognitive decline (modest for mild and significant for major) and may be attributed to Alzheimer’s, Parkinson’s, or cerebrovascular disease, a traumatic brain injury, frontotemporal lobar degeneration, dementia with Lewy Bodies, or other etiologies, but major neurocognitive disorders interfere with an individual’s ability to independently complete activities of daily living [10]. In a literature review on patient characteristics associated with decisional incapacity, persons with Alzheimer’s Disease (categorically separate from Mild Cognitive Impairment) were found to lack DMC over half (54%) of the time [11], underscoring the importance of DMC evaluation in the aging population.

## Construction and content

Aside from clinical interviews, numerous tools have been created to evaluate explicit capacities, albeit with differing approaches. With respect to specific DMCs, we found in a review of recent literature that the DMC to consent to medical treatment received the greatest attention. It was also the impetus for development of Appelbaum and Grisso’s four-ability model involving the abilities a) to *understand* information relevant to the decision, b) to *appreciate* the situation and the likely consequences of the available options, c) to *reason* and weigh the options against each other, based on one’s own values, and d) to make and *communicate* a choice [12, 13]. Interestingly, despite its wide acceptance across the world, the four-ability model has little support for use in forensic (medico-legal) settings [14], and it had been widely criticized for not focusing more on emotions and values [15]. Nonetheless, it remains the basis for many evaluation measures, including the MacCAT-T and the MacArthur Competence Assessment Tool for Clinical Research (MacCAT-CR). In addition to the MacCAT-T, recent research has documented 19 separate instruments just for the evaluation of DMC to consent to treatment, many of which had limited criterion validity and inter-rater reliability [16]. In 1977, Roth and colleagues suggested the most useful tools for DMC evaluation would be those that can be reliably applied, are accepted by more than one discipline, adequately balance autonomy with the need for treatment, and use objective rather than inferred or subjective means [13]. The field of neuropsychology can provide helpful assessments of certain cognitive aspects of DMC that can be used as part of a multidisciplinary evaluation, particularly as research has shown that DMC correlates more strongly with cognitive functioning than with psychiatric functioning [7].

In Switzerland, a recent survey indicated that most participating physicians only use unstructured clinical interviews to evaluate medical DMC [17]. Thereafter, a two-page DMC interview guide termed U-Doc [18] was developed based upon the four-ability model and adopted by the Swiss Academy of Medical Sciences in their 2018 guidelines on the evaluation of DMC [19]. The instrument does not comprise mandatory neuropsychological testing but emphasizes that it can be included in the evaluation procedure if necessary.

In health care, it is often the case that the treating physician is both the professional questioning a patient’s DMC and subsequently evaluating it. While some scholars cite the longstanding doctor-patient relationship and the primary physician’s knowledge of the patient’s history as important in a DMC evaluation [20], this approach tends to overestimate a patient’s DMC with low inter-rater reliability [21], and creates a dual role that could lead to conflicts of interest and violation of ethical standards for professional conduct [22]. While the dual role has the advantage of knowledge of the patient over time, presumably with an inherent internal measure of proportionality, low inter-rater reliability among health care professionals is something that has plagued DMC evaluation in general, whether the evaluators are compared with each other or with experts in the field [16, 23]. Additionally, there is limited consistency in how professionals evaluate DMC, and low agreement between subjective impressions of DMC and standardized evaluation procedures [24].

The lack of inter-rater reliability appears to be linked to the inherently value-laden nature of capacity assessments. Ethicists have cautioned that capacity evaluation is never fully neutral and that the personal values of the evaluators play a major role and should be considered [24]. Evaluations should be functional and specific to the decision at hand rather than a global determination because DMC may fluctuate and the requisite level of abilities depend on the complexity of the decision [24]. While it cannot be said that neuropsychological evaluations are not influenced to some degree by the evaluator, they can offer objective data that go beyond subject patient answers on given questions or mapping a patient’s response onto a particular aspect of DMC.

## Utility and discussion

The authors propose using a combination of traditional DMC assessment methods typically utilized by primary care physicians and attorneys, such as interviews and tools designed for specific capacities, with neuropsychological evaluation. This is particularly important in complicated cases and in cases where an individual’s underlying cognitive abilities are not well-known. As an example, the American Bar Association and the American Psychological Association took the stance many years ago that an evaluation of an individual’s capacity to carry out activities of daily living must include an assessment of the underlying cognitive and judgment skills to be sufficient [25]. Cognitive impairment related to dementia is the most common threat to living independently for adults and an evaluation of the capacity to live independently should include measures of attention and orientation, episodic memory, executive functioning, and language skills, which are considered to be the cognitive underpinnings of this capacity [25]. This connection is not limited to activities of daily living either. Specific cognitive domains are considered to underly the capacity to create and execute a will, including semantic knowledge, autobiographical memory, recent memory, working memory and other executive functions, as well as language [6, 14]. An interdisciplinary team approach involving a patient’s physicians and specialists, their attorney, and a neuropsychologist would allow for the most thorough and ethical evaluation of a patient’s DMC whereby a neuropsychologist can assess underlying skills and provide information as to how any limitations might impact a patient’s DMC in medicolegal settings. This would provide physicians, including specialists such as neurologists, and attorneys with critical information to use in making DMC determinations.

Importantly, a neurocognitive model of medical DMC has been proffered as the best way to understand this particular capacity in an aging population with memory impairments. The model is based upon three core cognitive tasks involved in a patient’s determination of treatment preferences, including “(a) comprehension, encoding and retrieval of treatment information; (b) information processing and internally arriving at a treatment decision; and (c) verbal or written communication of the treatment decision” [26 p223]. The authors suggest administering measures of receptive language, short-term memory, executive functioning, and judgment/reasoning to address the three core cognitive tasks [26]. The use of interviews or specific capacity measures alone does not give a practitioner information about the presence or absence of impairment in underlying cognitive abilities. It is for this reason that physicians, lawyers, and others involved in DMC evaluation should seek neuropsychological consultation in complicated cases and cases where there are questions about specific cognitive domains such as memory or attention,

## Conclusions

Although the face validity associated with tools designed to evaluate specific DMCs is appealing, questions remain as to whether or not the degree of difficulty and test performance can be generalized to a real-life situation [23]. Additionally, these kinds of instruments do not capture the underlying cognitive abilities that subserve any given capacity [23]. Therefore, in certain cases, neurocognitive testing can and should be used to provide valuable additional information for the DMC evaluation. Such two-part evaluations involving a neuropsychological exam and specific capacity-related inquiries is offered as a way of increasing standardization, providing structure, and limiting subjectiveness in the evaluation [3, 23]. While it may be neither feasible nor desirable for every capacity evaluation, it should be an approach for complicated cases despite its inability to completely eliminate the subjectiveness in DMC evaluation, which is never a purely descriptive task but inherently involves normative deliberation by the evaluating person [24].

## Data Availability

Data sharing is not applicable to this article as no datasets were generated or analyzed during the current study.

## Abbreviations

DMC: Decision-Making Capacity
ACP: Advance Care Planning
MacCAT-T: MacArthur Competence Assessment Tool for Treatment
CERAD-NAB: Consortium to Establish a Registry for Alzheimer’s Disease – Neuropsychological Assessment Battery
MMSE: Mini Mental Status Exam
MoCA: Montreal Cognitive Assessment
